# Novel Genetic Signatures Associated With Sporadic Amyotrophic Lateral Sclerosis

**DOI:** 10.3389/fgene.2022.851496

**Published:** 2022-03-24

**Authors:** Robert Logan, Juleah Dubel-Haag, Nicolas Schcolnicov, Sean J. Miller

**Affiliations:** ^1^ Pluripotent Diagnostics Corp, Colorado Springs, CO, United States; ^2^ Department of Biology, Eastern Nazarene College, Quincy, MA, United States

**Keywords:** amyotrophic lateral sclerosis, genetics, dementia, Ndufs4, mitochondria, NADH dehydrogenase

## Abstract

Amyotrophic Lateral Sclerosis (ALS) is a complex polygenetic neurodegenerative disorder. Establishing a diagnosis for ALS is a challenging and lengthy process. By the time a diagnosis is made, the lifespan prognosis is only about two to 5 years. Genetic testing can be critical in assessing a patient’s risk for ALS, provided they have one of the known familial genes. However, the vast majority of ALS cases are sporadic and have no known associated genetic signatures. Our analysis of the whole genome sequencing data from ALS patients and healthy controls from the Answer ALS Consortium has uncovered twenty-three novel mutations in twenty-two protein-coding genes associated with sporadic ALS cases. The results show the majority of patients with the sporadic form of ALS have at least one or more mutation(s) in the 22 genes we have identified with probabilities of developing ALS ranging from 25–99%, depending on the number of mutations a patient has among the identified genes. Moreover, we have identified a subset of the ALS cohort that has >17 mutations in the 22 identified. In this case, a patient with this mutation profile has a 99% chance of developing ALS and could be classified as being at high risk for the disease. These genetic biomarkers can be used as an early ALS disease diagnostic tool with a rapid and non-invasive technique.

## Introduction

An earlier diagnosis allows for earlier therapeutic intervention, which is critically needed for neurodegenerative diseases (Pan et al., 2020; Logan et al., 2021a; Logan et al., 2021b). ALS is a uniquely difficult disease to diagnosis (Singh et al., 2019). There is considerable variety in the disease pathology and progression by the time patients seek out medical care (Miller et al., 2017; Miller, 2018; Miller et al., 2018; Bendotti et al., 2020). Additionally, an early ALS diagnosis is challenging to make because it can mask as several other movement disorders (Bosco et al., 2011). The process of establishing an ALS diagnosis is largely based on excluding differential diagnoses (Campanari et al., 2019). These complications result in a late diagnosis, after the underlying pathology is already well underway. The universal prognosis of about two to 5 years left to live post-diagnosis is evidence of this fact (Knibb et al., 2016; Su et al., 2021). There is a dire need to establish an earlier diagnosis of ALS so that those with the disease can have a fighting chance at earlier therapeutic intervention.

ALS cases can be grouped by two categories: familial ALS (fALS), where the patient has a genetically related family member also affected, and sporadic ALS (sALS), where the patient has no family history of ALS (Zhang et al., 2016; Mejzini et al., 2019). Historically, 5–10% of cases are fALS, and the other 90–95% cases are sALS (Boylan, 2015). In the past 10 years, the C9ORF72 hexanucleotide repeat expansion, has been identified as the most prevalent genomic mutation found in the ALS disease population (Donnelly et al., 1016).

C9ORF72 repeat expansions can be found in up to 34% of fALS and 5% of sALS cases. High associations with C9ORF72 to the pathologically related neurodegenerative disorder, Frontotemporal Dementia (FTD), have also been shown (Donnelly et al., 1016).

Genetic testing for disease risk can occur before the onset of symptoms and is becoming increasingly cheap, efficient, and accurate (Germain et al., 2021). The currently known genes associated with ALS are largely restricted to the 10% of fALS, leaving many of the 90% of sALS patients without a reliable genetic screening assay (Kim et al., 2020). Moreover, it is currently believed that ALS is a polygenic disease, meaning that more than one gene is involved in the disease (Bandres-Ciga et al., 2019). Currently there have been over 30 genes identified with the development of ALS and several hundreds of genetic mutations have been implicated in ALS, with the majority of the genes identified being in the familial form of the disease (Kim et al., 2020). Because current genetic tests focus on genes associated with the familial form of the disease, patients with the sporadic form of the disease are left with little hope identifying the disease before symptoms occur.

We have identified 22 genes, that when mutated, are highly associated with the development of the sporadic form of ALS. These genetic mutations have not been correlated with ALS before, although their protein products are often involved in pathways that are dysregulated in ALS (Fogarty et al., 2015). For example, mutations in the mitochondrial protein-coding gene, NDUFS4, are found in >30% of the ALS patients in our study. Furthermore, none of these genetic mutations are identified in the healthy controls. We have identified these novel ALS-associated genetic mutations using the latest cohort data from Answer ALS, which is the largest research partnership currently in existence focused on ALS (Berry et al., 2001). We have been able to derive a probability ranking for having ALS based on a patient’s genetic profile of these genes.

## Materials and Methods

Whole genome sequencing (WGS) data from 713 ALS patients (484 males, 322 females) and 93 healthy controls (63 males and 30 females) were provided by the Answer ALS consortium. In order to establish a class balance, a total of 818 (491 males, 327 females) additional healthy control samples were used from the Alzheimer’s Disease Neuroimaging Initiative genetic dataset, ensuring the controls were neurotypical. Patients were tested for familial genes (c9orf72, SOD1, TDP43) if family history of ALS was present; 542 of the 713 ALS patients were sporadic.

Clinical data from the Answer ALS consortium (https://www.answerals.org) is coordinated through Massachusetts General Hospital and Johns Hopkins University. The eight neuromuscular clinics responsible for enrolling patients included Johns Hopkins University, Massachusetts General Hospital, Ohio State, Emory University, Washington University, Northwestern University, Cedars Sinai and Neurology Group at Texas. These institutions were selected based on their geographical location as well as their expertise in ALS research. The study was approved by the institutional review board associated with each of the eight institutions. These include the Johns Hopkins Medicine Institutional Review Board, the Mass General Brigham Human Research Committee, the Biomedical IRB of Ohio State, the Emory University Institutional Review Board, the Washington University in St. Louis’s Institutional Review Board, the Northwestern University Institutional Review Board, the Cedars-Sini Institutional Review Board, and the Texas Neurology Institutional Review Board. All participants were provided with the same written informed consent prior to undergoing any study procedures. Participants were provided with a global unique identifier (NeuroGUID) and were monitored every 3 months for an entire year. All of the methods employed in this study were performed in accordance with the relevant guidelines and regulations, including the Declaration of Helsinki (Berry et al., 2001; Rothstein et al., 2020).

The additional control data used from the Alzheimer’s Disease Neuroimaging Initiative (ADNI) database (adni.loni.usc.edu) was regulated, collected, and disseminated according to the ADNI investigator plans for study design and implementation. However, ADNI investigators had no part in this currently presented report, including the analysis or writing. A complete listing of ADNI Infrastructure Investigators can be found at: http://adni.loni.usc.edu/wp-content/uploads/how_to_apply/ADNI_Acknowledgement_List.pdf.

DNA was extracted from peripheral blood mononuclear cells (PBMCs) at the New York Genome Center (NYGC) using the standard protocol of the ChargeSwitch gDNA Serum Kit (Thermo Fisher Scientific, Waltham, MA). Subsequently, library preparation and WGS was performed using Illumina HiSeq X10 (2Å∼150 paired-end reads). Paired end reads were aligned to the GRCh38 human reference using the Burrows-Wheeler Aligner (BWAMEMv0.7.8)^21^.

Aligned reads were then processed using the GATK best-practices workflow, which includes the marking of duplicate reads with Picard tools (v1.83, http://broadinstitute.github.io/picard/), local realignment around indels, and base quality score recalibration (BQSR) *via* Genome Analysis Toolkit (GATK v3.5.0). Variant calls were made using GATK’s HaplotypeCaller walker, and variant quality score recalibration (VQSR) was performed. Variant calling was performed at NYGC. Variants (heterozygous or homozygous) present in at least 1/713 affected ALS patients and 0/93 healthy controls samples were identified. The remaining variants were annotated using SnpEff and filtered based on functional impacted to extract variants with predicted effects on protein coding.

Upper and lower bounds were set on the true fractions of either the ALS population or the control population, with a given mutation. The number of people within a group that are positive for the mutation-of-interest will have a binomial distribution. Clopper-Pearson intervals on the binomial proportion are calculated for true population proportions. The probability (*p*-value) of the null hypothesis that a mutation is present in the ALS population in the same proportion as in the control population. Fisher’s exact tests are approximations of this probability which converge to the exact value in the limit of large group size.

## Results

### Variants in the Coding-Genome Found Only in ALS Patients

Identification of patients in the ALS population that contain variants that are not present in the healthy control population renders valuable insight into disease pathogenesis and genetic diagnostics. After sorting through the entire cohort of ALS patients and the healthy control samples, 542 of the 713 ALS patients were sporadic, the remaining familial cases were excluded from this study. We found that we could detect 44, 156, 401 variants present only in the ALS population.

C9ORF72 hexanucleotide-repeats are the most prevalent ALS mutation know to data, affecting 5–10% of all cases and up to 34% of familial (fALS). Of the detect variants in the ALS-only samples, we focused on the variants in greater than 22% of the ALS population to determine candidate genes in a higher than previously accepted association. We found that there were 23 variants in 22 different genes that reached this level of significance, with a *p*-value of less than 2.2 × 10^–16^, all of which are more significant than C9ORF72 reported population (Table 1). Top variants of interest and their significance are shown.

**TABLE 1:** Shows the 22 genes that are not mutated in the control sample. The gene names, the number of ALS cases out of the 713-patient cohort, percent of total ALS cases with the 99% CL Clopper-Pearson interval are shown, and *p*-value, respectively.

Gene name	Cases (713)	% ALS cases	Fisher exact
NDUFS4	228	$31.98_{-4.44}^{+4.68}$	<2.2e-16
AC106707.1	216	$30.29_{-4.35}^{+4.63}$	<2.2e-16
ZC3H7B	215	$30.15_{-4.35}^{+4.63}$	<2.2e-16
AC023095.1	209	$29.31_{-4.31}^{+4.59}$	<2.2e-16
CCDC59	209	$29.31_{-4.31}^{+4.59}$	<2.2e-16
TXNP1-INPP5F (1)	208	$29.17_{-4.3}^{+4.59}$	<2.2e-16
TXNP1-INPP5F (2)	207	$29.03_{-4.29}^{+4.58}$	<2.2e-16
TNRC18	205	$28.75_{-4.28}^{+4.57}$	<2.2e-16
TOP2A	203	$28.47_{-4.27}^{+4.56}$	<2.2e-16
THRAP3	202	$28.33_{-4.26}^{+4.55}$	<2.2e-16
TRPM3	202	$28.33_{-4.26}^{+4.55}$	<2.2e-16
ATP10A	202	$28.33_{-4.26}^{+4.55}$	<2.2e-16
FAM184B	201	$28.19_{-4.25}^{+4.55}$	<2.2e-16
AC096747.1-NDUFB5P1	201	$28.19_{-4.25}^{+4.55}$	<2.2e-16
NCS1	200	$28.05_{-4.24}^{+4.54}$	<2.2e-16
AC007690.1	199	$27.91_{-4.24}^{+4.54}$	<2.2e-16
AL033528.3	197	$27.63_{-4.22}^{+4.52}$	<2.2e-16
RN7SL33P	197	$27.63_{-4.22}^{+4.52}$	<2.2e-16
COX5A	195	$27.35_{-4.21}^{+4.51}$	<2.2e-16
AL161629.1	167	$23.42_{-3.96}^{+4.33}$	<2.2e-16
SLF1	164	$23_{-3.94}^{+4.31}$	<2.2e-16
LIPH	163	$22.86_{-3.93}^{+4.3}$	<2.2e-16
RPL5P16-AC008885.1	153	$21.46_{-3.83}^{+4.21}$	<2.2e-16

### Genomic Mutations in Genes of the ALS-Only Population

We identified variants at a gene level in the ALS population and not in healthy control. We sorted and analyzed candidate genes based on the presence of at least one variant rather than individual variants. This approach would allow us to identify gene-modifiers involved in ALS pathology. We found 22 individual genes that were each mutated in over 21% of the ALS cases (Table 1). One gene, NDUFS4 (NADH Dehydrogenase Iron-Sulfur Protein 4), was mutated in over 30% of the ALS population. The high proportion of ALS patients with variants in the 22 identified genes is suggestive that the mutations in the 22 identified genes are associated with ALS disease in a large proportion of patients.

### Clinical Evaluation of ALS Patients

Multiple clinical phenotypes can be associated with the onset of ALS, including age, sex, and scoring from the Revised ALS Functional Rating Scale (ALSFRS-R). We compared the overall age and sex of onset for the entire ALS population and the patients we can diagnose with our classifier analysis. There are no statistically significant differences detected in the age, sex, or ALSFRS-R for our diagnosable patients compared to the overall ALS population (Supplementary Figures 1A,B, Supplementary Material S1).

Next, we compared the three different areas of disease onset, “axial,” “bulbar,” and “limb,” and each potential correlation to our candidate genetic mutations. We found no significant difference between the clinical appearance of disease onset in our top gene candidate patients and the symptoms displayed in the overall ALS population (data not shown).

### Classifier Analysis for the Top Candidate Genes in the ALS-Only Population

Diagnostic testing based on novel gene sequence identification could serve as an early disease detection tool. We designed a classifier analysis to determine if our top 22 gene candidates, single or in combination, could be used as a statistical tool to associate and identify the ALS-only population. Evaluation of the top 22 candidates led to the discovery that majority of the ALS samples had at least one or two of our 23 specific loci mutated and peaked 17–20 loci and 22–23 loci, respectively. Our results show that the sensitivity of detecting ALS was 58.62% ± 4.8% when at least one of the 23 loci was mutated, and 40.4% ± 4.7% at 99% CL when at least two of the 23 loci was mutated, with a specificity of 100% because none of the 22 genes or 23 loci are mutated in the normal population. Our results also show that the probability of detecting ALS increases as the number of positive test results for the 23 loci increase (Table 2).

**TABLE 2:** Shows the sensitivity and specificity of combined loci in detecting ALS. The sensitivity of any number of combination of mutations and specificity are shown.

Number of mutations	Sensitivity (%)	Specificity (%)
1	$58.62_{-4.8}^{+4.8}$	100
2	$40.4_{-4.7}^{+4.7}$	100
3	$34.08_{-4.6}^{+4.6}$	100
4	$32.26_{-4.5}^{+4.5}$	100
5	$31.56_{-4.5}^{+4.5}$	100
6	$31.28_{-4.5}^{+4.5}$	100
7	$31.14_{-4.5}^{+4.5}$	100
8	$31_{-4.5}^{+4.5}$	100
9	$31_{-4.5}^{+4.5}$	100
10	$30.86_{-4.5}^{+4.5}$	100
11	$30.86_{-4.5}^{+4.5}$	100
12	$30.86_{-4.5}^{+4.5}$	100
13	$30.86_{-4.5}^{+4.5}$	100
14	$30.86_{-4.5}^{+4.5}$	100
15	$30.72_{-4.5}^{+4.5}$	100
16	$30.3_{-4.4}^{+4.4}$	100
17	$28.19_{-4.4}^{+4.3}$	100
18	$25.39_{-4.2}^{+4.2}$	100
19	$20.34_{-3.9}^{+3.9}$	100
20	$15_{-3.4}^{+3.4}$	100
21	$8.56_{-2.7}^{+2.7}$	100
22	$2.66_{-1.6}^{+1.6}$	100
23	$0.42_{-0.6}^{+0.6}$	100

**FIGURE 1:**
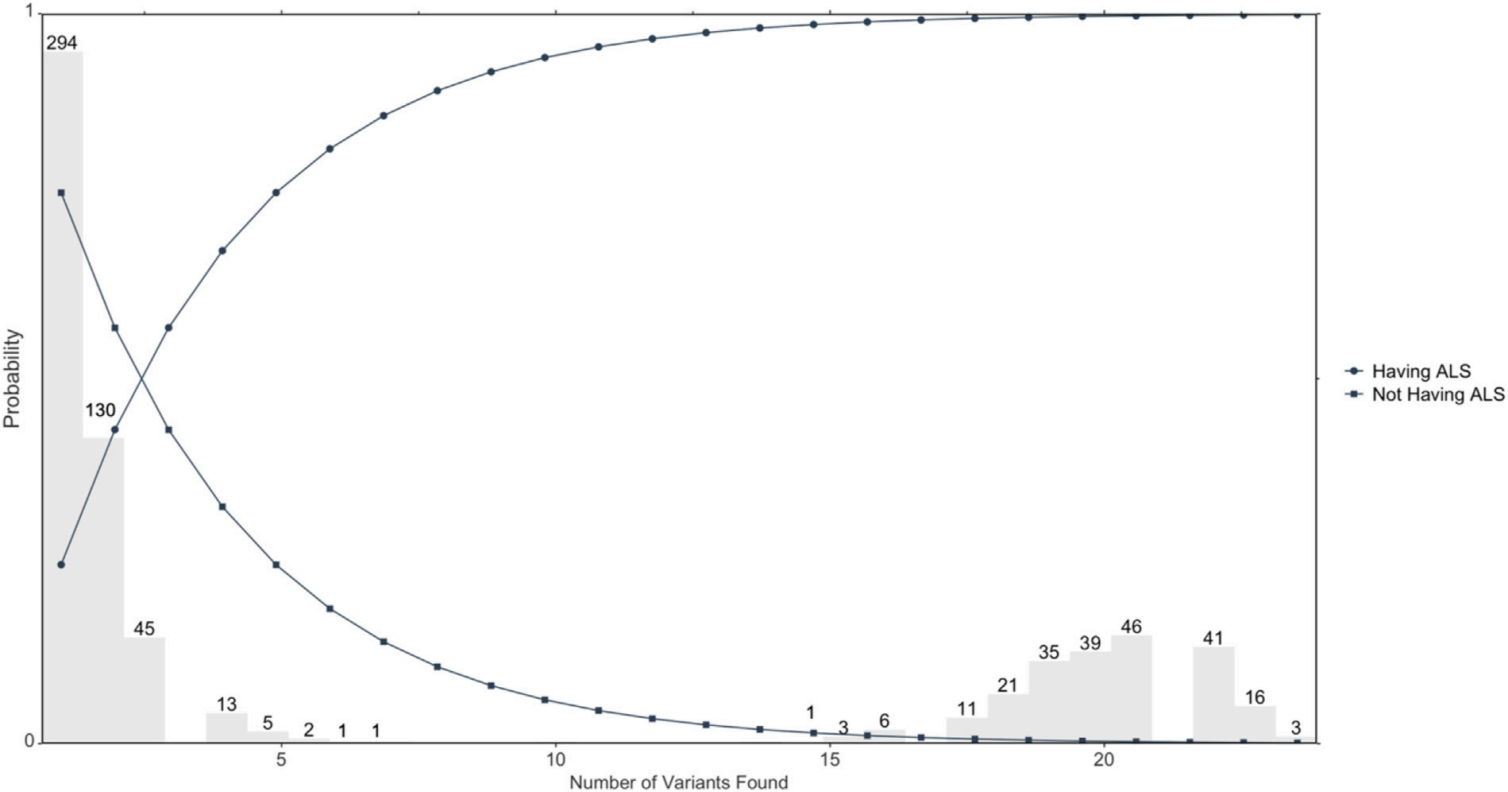
Illustrates the probability of developing ALS based on the number of variants mutated. Circles represent the probability of developing ALS determined by the number of overall variants that are found; *n* = 713 ALS cases.

Figure 1 illustrates distribution of candidate ALS-only mutated genes and probability of having ALS or not having ALS based on the number of positive results or negative results of mutations. The distribution of numbers of variants found out of the 23 genomic loci in the 713 ALS cases is shown in grey. The circle represents the probability of having ALS, which shows an increasing probability with increasing positive numbers of variants. Further, the square represents the probability of not having ALS, which shows a decreasing probability with increasing positive numbers of variants.

Our results show that the majority of patients with the sporadic form of ALS have at least one or more mutation(s) in the 22 genes that we have identified, with probabilities of developing ALS ranging from 25–99% depending on the number of mutations a patient has among the identified genes. Moreover, we have identified a subset of the ALS cohort that has >17 mutations in the 22 identified. In this case, a patient with this mutation profile has a 99% chance of developing ALS and could be classified as being at high risk for the disease.

When evaluating the potential for multifactorial genetic involvement in ALS patients with a classifier analysis using our top 22 gene candidates. We found over 50% of ALS cases with at least seventeen of our 22 candidate genes mutated, can be predicted with a false-positive rate of less than 0.1%, at 99% confidence level (CL). This establishes that a majority of our ALS patients have mutations in at least seventeen of our 22 candidate genes.

## Discussion

This data demonstrates variants in coding-regions or entire genes that are associated in a majority of the ALS population (Mejzini et al., 2019). In this clinical and biomedical trial, the Answer ALS consortium utilized the latest next-generation sequencing technology and annotation with the highest quality control and protocols to allow us to perform unbiased genetic analyses on protein-coding genes and other genomic areas of interest (Berry et al., 2001). We are the first to report on this novel genomic database using these statistical and computational methods.

We designed an analysis focused on identification of variants in the coding-genome. This model allowed us to detect individual SNPs in 27% of the ALS cohort. When using a gene-level approach, we robustly identify a majority (>50%) of the ALS population at 79% CL using a one-sided Clopper-Pearson interval. Other known ALS-linked genes each consist of less than 10% of the overall ALS population.

The identification of NDUFSA as a candidate gene mutated in 27% of the ALS group raises support for prior disease biological findings, such as the influence of SOD1 mutations. SOD1, was first reported to be associated with ALS in 1993, and since has been one of the most well-studied ALS-linked genes. SOD1 plays essential roles in cellular antioxidant defense mechanisms (Mejzini et al., 2019). There have been over 150 discovered mutations in SOD1, with each representing a different level of severity in ALS pathogenesis (Rothstein et al., 1995).

Similar to SOD1, NDUFS4, is another essential member of the antioxidant defense mechanisms in the cell. Recent literature has shown that NDUFS4 knockout in *Drosophila melanogaster (Dmel)* neurons and muscle significantly reduced their lifespan (Foriel et al., 2018). The Tabula-Muris database reports that NDUFS4 is mainly expressed in neurons and muscle cells (Muris, 2018). These supportive findings of our top gene candidate in NDUFS4 are further evidence that NDUFS4 should be explored in genetic screening, and may lend insight into how specific mutations may result in abnormal protein function in ALS (Quintana et al., 2010).

Secondly, our data revealed that the Neuronal Calcium Sensor-1 (NCS-1) gene is mutated in a large population of sporadic ALS patients. Decades of research have been heavily focused on the role of neuronal calcium signaling in motor neurons of ALS patients (Tedeschi et al., 2019). Other movement disorders, such as Parkinson’s disease have shown the influence of NCS-1 in neuronal activity of dopaminergic neurons (Dragicevic et al., 2014). This raises an important question and avenue for future functional experimentation to identify if NCS-1 is implemented in ALS pathogenesis.

Pathway analysis programs such as PANTHER have aided studies in the identification of associations in protein-coding genes (Mi et al., 2021). We applied gene ontology (GO) enrichment analysis with our list of genes associated with sporadic ALS. Surprisingly, no statistical significance results were found in our GO performance (Gaudet and Dessimoz, 2017). This potentially highlights the caveats with GO, where bias has been reported towards 16% of the human genome, leaving genetic relationships with a majority lacking (Tomczak et al., 2018).

The limitations in this study call for increased awareness and participation in clinical trials (Kiernan et al., 2021). This study including all of the patients in the Answer ALS cohort but lacked the appropriate amount of healthy control samples. To avoid oversampling, we applied a method to incorporate the cognitively normal healthy controls from ADNI (Whalen et al., 2021). Nonetheless, until additional ALS and healthy patients become available for replicative cohorts, this study remains of high importance for the identification of correlated sporadic ALS genes.

The ability to detect and diagnose ALS before clinical- and pathological-onset is imperative to prolonging patient lifespan, understanding the pathobiology, and designing therapies for early intervention. ALS is a devastating neurodegenerative disorder, with no cures or genetic diagnostics. We now report the detection of >30% of the ALS-only population, at 99% CL, can be achieved with our candidate genomic signatures. This is the first report to find novel genetic targets associated with such a high level of the ALS community. These findings are optimistic for the use of genetic screening in early ALS diagnosis and therapeutic intervention.

## Data Availability

The datasets presented in this study can be found in online repositories. The names of the repository/repositories and accession number(s) can be found in the article/Supplementary Material.

## References

[B1] Bandres-Ciga S. Noyce A. J. Hemani G. Nicolas A. Calvo A. Mora G. (2019). Shared Polygenic Risk and Causal Inferences in Amyotrophic Lateral Sclerosis. Ann. Neurol. 85, 470–481. 10.1002/ana.25431 30723964 PMC6450729

[B2] Bendotti C. Bonetto V. Pupillo E. Logroscino G. Al-Chalabi A. Lunetta C. (2020). Focus on the Heterogeneity of Amyotrophic Lateral Sclerosis. Amyotroph. Lateral Scler. Frontotemporal Degeneration 21, 485–495. 10.1080/21678421.2020.1779298 32583689

[B3] Berry J. Thompson T. Li J. Kaye J. A. Lim R. G. Wu J. (2001). Answer ALS : A Large-Scale Resource for Sporadic and Familial ALS Combining Clinical Data with Multi-Omics Data from Induced Pluripotent Cell. Nat. Neurosci. 25, 1–31. 10.1038/s41593-021-01006-0 PMC8825283 35115730

[B4] Bosco D. A. LaVoie M. J. Petsko G. A. Ringe D. (2011). Proteostasis and Movement Disorders: Parkinson's Disease and Amyotrophic Lateral Sclerosis. Cold Spring Harb. Perspect. Biol. 3, a007500–24. 10.1101/cshperspect.a007500 21844169 PMC3179340

[B5] Boylan K. (2015). Familial Amyotrophic Lateral Sclerosis. Neurol. Clin. 33, 807–830. 10.1016/j.ncl.2015.07.001 26515623 PMC4670044

[B6] Campanari M. L. Bourefis A. R. Kabashi E. (2019). Diagnostic challenge and Neuromuscular junction Contribution to ALS Pathogenesis. Front. Neurol. 10, 68–8. 10.3389/fneur.2019.00068 30787905 PMC6372519

[B7] Donnelly C. J. Zhang P. W. Pham J. T. Haeusler A. R. Mistry N. A. Vidensky S. . RNA Toxicity from the ALS/FTD C9ORF72 Expansion Is Mitigated by Antisense Intervention. Neuron 79, 415–428. 10.1016/j.neuron.2013.10.015 PMC4098943 24139042

[B8] Dragicevic E. Poetschke C. Duda J. Schlaudraff F. Lammel S. Schiemann J. (2014). Cav1.3 Channels Control D2-Autoreceptor Responses via NCS-1 in Substantia Nigra Dopamine Neurons. Brain 137, 2287–2302. 10.1093/brain/awu131 24934288 PMC4107734

[B9] Fogarty M. J. Noakes P. G. Bellingham M. C. (2015). Motor Cortex Layer V Pyramidal Neurons Exhibit Dendritic Regression, Spine Loss, and Increased Synaptic Excitation in the Presymptomatic hSOD1G93A Mouse Model of Amyotrophic Lateral Sclerosis. J. Neurosci. 35, 643–647. 10.1523/jneurosci.3483-14.2015 25589758 PMC6605367

[B10] Foriel S. Beyrath J. Eidhof I. Rodenburg R. J. Schenck A. Smeitink J. A. M. (2018). Feeding Difficulties, a Key Feature of the Drosophila NDUFS4 Mitochondrial Disease Model. Dis. Model. Mech. 11. 10.1242/dmm.032482 PMC5897729 29590638

[B11] Gaudet P. Dessimoz C. (2017). Gene Ontology: Pitfalls, Biases, and Remedies. Methods Mol. Biol. 1446, 189–205. 10.1007/978-1-4939-3743-1_14 27812944

[B12] Germain D. P. Moiseev S. Suárez-Obando F. Al Ismaili F. Al Khawaja H. Altarescu G. (2021). The Benefits and Challenges of Family Genetic Testing in Rare Genetic Diseases—Lessons from Fabry Disease. Mol. Genet. Genomic Med. 9, 1–16. 10.1002/mgg3.166 PMC8172211 33835733

[B13] Kiernan M. C. Vucic S. Talbot K. McDermott C. J. Hardiman O. Shefner J. M. (2021). Improving Clinical Trial Outcomes in Amyotrophic Lateral Sclerosis. Nat. Rev. Neurol. 17, 104–118. 10.1038/s41582-020-00434-z 33340024 PMC7747476

[B14] Kim G. Gautier O. Tassoni-Tsuchida E. Ma X. R. Gitler A. D. (2020). ALS Genetics: Gains, Losses, and Implications for Future Therapies. Neuron 108, 822–842. 10.1016/j.neuron.2020.08.022 32931756 PMC7736125

[B15] Knibb J. A. Keren N. Kulka A. Leigh P. N. Martin S. Shaw C. E. (2016). A Clinical Tool for Predicting Survival in ALS. J. Neurol. Neurosurg. Psychiatry 87, 1361–1367. 10.1136/jnnp-2015-312908 27378085 PMC5136716

[B16] Logan R. Williams B. G. Ferreira da Silva M. Indani A. Schcolnicov N. Ganguly A. (2021). Deep Convolutional Neural Networks with Ensemble Learning and Generative Adversarial Networks for Alzheimer’s Disease Image Data Classification. Front. Aging Neurosci. 13, 1–12. 10.3389/fnagi.2021.720226 PMC8416107 34483890

[B17] Logan R. Zerbey S. S. Miller S. J. (2021). The Future of Artificial Intelligence for Alzheimer's Disease Diagnostics. Aad 10, 53–59. 10.4236/aad.2021.104005

[B18] Mejzini R. Flynn L. L. Pitout I. L. Fletcher S. Wilton S. D. Akkari P. A. (2019). ALS Genetics, Mechanisms, and Therapeutics: Where Are We Now. Front. Neurosci. 13, 1310–1327. 10.3389/fnins.2019.01310 31866818 PMC6909825

[B19] Mi H. Ebert D. Muruganujan A. Mills C. Albou L.-P. Mushayamaha T. (2021). PANTHER Version 16: A Revised Family Classification, Tree-Based Classification Tool, Enhancer Regions and Extensive API. Nucleic Acids Res. 49, D394–D403. 10.1093/nar/gkaa1106 33290554 PMC7778891

[B20] Miller S. J. (2018). Astrocyte Heterogeneity in the Adult central Nervous System. Front. Cel. Neurosci. 12, 1–6. 10.3389/fncel.2018.00401 PMC6262303 30524236

[B21] Miller S. J. Glatzer J. Hsieh Y. Rothstein J. (2018). Cortical Astroglia Undergo Transcriptomic Dysregulation in the G93A SOD1 ALS Mouse Model. J. Neurogenet. 32, 322–335. 10.1080/01677063.2018.1513508 30398075 PMC6444185

[B22] Miller S. J. Zhang P.-w. Glatzer J. Rothstein J. D. (2017). Astroglial Transcriptome Dysregulation in Early Disease of an ALS Mutant SOD1 Mouse Model. J. Neurogenet. 31, 37–48. 10.1080/01677063.2016.1260128 28019127

[B23] Muris T. (2018). 乳鼠心肌提取 HHS Public Access. Nature 562, 362–372. 10.1186/2046-4053-4-1

[B24] Pan D. Zeng A. Jia L. Huang Y. Frizzell T. Song X. (2020). Early Detection of Alzheimer's Disease Using Magnetic Resonance Imaging: A Novel Approach Combining Convolutional Neural Networks and Ensemble Learning. Front. Neurosci. 14, 259. 10.3389/fnins.2020.00259 32477040 PMC7238823

[B25] Quintana A. Kruse S. E. Kapur R. P. Sanz E. Palmiter R. D. (2010). Complex I Deficiency Due to Loss of Ndufs4 in the Brain Results in Progressive Encephalopathy Resembling Leigh Syndrome. Proc. Natl. Acad. Sci. 107, 10996–11001. 10.1073/pnas.1006214107 20534480 PMC2890717

[B26] Rothstein J. D. Van Kammen M. Levey A. I. Martin L. J. Kuncl R. W. (1995). Selective Loss of Glial Glutamate Transporter GLT-1 in Amyotrophic Lateral Sclerosis. Ann. Neurol. 38, 73–84. 10.1002/ana.410380114 7611729

[B27] Rothstein J. Thompson T. Li J. Kaye J. A. Lim R. G. Wu J. (2020). Answer ALS: A Large-Scale Resource for Sporadic and Familial ALS Combining Clinical Data with Multi-Omics Data from Induced Pluripotent Cell Lines. Nat. Neurosci. 25, 226. 10.1038/s41593-021-01006-0 PMC8825283 35115730

[B28] Singh N. Ray S. Srivastava A. (2019). Clinical Mimickers of Amyotrophic Lateral Sclerosis-Conditions We Cannot Afford to Miss. Ann. Indian Acad. Neurol. 22, 351. 10.4103/aian.AIAN_456_18 PMC6613410 31359959

[B29] Su W.-M. Cheng Y.-F. Jiang Z. Duan Q.-Q. Yang T.-M. Shang H.-F. (2021). Predictors of Survival in Patients with Amyotrophic Lateral Sclerosis: A Large Meta-Analysis. EBioMedicine 74, 103732. 10.1016/j.ebiom.2021.103732 34864363 PMC8646173

[B30] Tedeschi V. Petrozziello T. Secondo A. (2019). Calcium Dyshomeostasis and Lysosomal Ca2+ Dysfunction in Amyotrophic Lateral Sclerosis. Cells 8, 1216. 10.3390/cells8101216 PMC6829585 31597311

[B31] Tomczak A. Mortensen J. M. Winnenburg R. Liu C. Alessi D. T. Swamy V. (2018). Interpretation of Biological Experiments Changes with Evolution of the Gene Ontology and its Annotations. Sci. Rep. 8, 5115. 10.1038/s41598-018-23395-2 29572502 PMC5865181

[B32] Whalen S. Schreiber J. Noble W. S. Pollard K. S. (2021). Navigating the Pitfalls of Applying Machine Learning in Genomics. Nat. Rev. Genet. 23, 169–181. 10.1038/s41576-021-00434-9 34837041

[B33] Zhang K. Donnelly C. J. Haeusler A. R. Grima J. C. Machamer J. B. Steinwald P. (2016). The C9orf72 Repeat Expansion Disrupts Nucleocytoplasmic Transport. Nature 525, 56–61. 10.1038/nature14973 PMC4800742 26308891

