# Expression of Concern: Novel Genetic Signatures Associated With Sporadic Amyotrophic Lateral Sclerosis

**DOI:** 10.3389/fgene.2022.946214

**Published:** 2022-05-25

**Authors:** Frontiers Editorial Office

With this notice, Frontiers states its awareness of serious complaints regarding this article. This expression of concern has been posted while Frontiers investigates the allegations. The situation will be updated as soon as the investigation is complete.

